# Acclimatization and Deacclimatization to Oxygen: Determining Exposure Limits to Avoid CNS O_2_ Toxicity in Active Diving

**DOI:** 10.3389/fphys.2020.01105

**Published:** 2020-09-04

**Authors:** Ran Arieli, Ben Aviner

**Affiliations:** ^1^The Israel Naval Medical Institute, Israel Defense Forces Medical Corps, Haifa, Israel; ^2^Eliachar Research Laboratory, Western Galilee Medical Center, Nahariya, Israel

**Keywords:** oxygen toxicity, central nervous system, diving, exercise, risk assessment

## Power Equation for Prediction of Central Nervous System Oxygen Toxicity

We have proposed the power equation as a measure of central nervous system oxygen toxicity (CNS-OT) (Arieli et al., 2002), and believe it has the best predictive power of any approach suggested to date. This algorithm was derived from 2,039 closed-circuit oxygen dives (1.2–1.6 bar, water temperature 17–28°C, data extracted by author R.A. from Israel Navy dive records) and 661 immersed exercising chamber exposures (1.6–2.5 bar, data extracted from the scientific literature), a total of 2,700 submerged exercise exposures at a mean metabolic rate of 4.4 metabolic equivalents of task (METs). CNS-OT occurred in 104 of these exposures. The various symptoms reported and considered positive evidence of CNS-OT were nausea, numbness, dizziness, twitching, hearing and visual disturbances, and convulsions. The percentage of symptomatic dives was 0% of dives conducted at 1.2 bar (*n* = 64); 2.5% of the dives at 1.3 bar (*n* = 711); 3.4% of those at 1.4 bar (*n* = 269); 4.0% of the dives at 1.5 bar (*n* = 1,108), and 6.3% of those conducted at 1.6 bar (*n* = 164) (Arieli et al., 2006). We found no previous reports of CNS-OT occurring at 1.3 bar, and we have no knowledge of any similar follow-up of novice closed-circuit oxygen divers, asking them to provide information on incidents which may have occurred in the course of their training. In our recent elaboration of this method (Arieli, 2019), we showed that the CNS-OT index K for exercise at 4.4 MET may be given by the equation: **K** **=** **t**^**2**^${\text{PO}}_{2}^{6.8}$, where t is the time in min and PO_2_ is the oxygen pressure in bar.

Recovery of the index K (Krec) was calculated by the equation:

**Krec** **=** **K×e**^**−0.079trec**^, where trec is the recovery time in min.

The appropriate risk (Z) can be calculated from the normal distribution using the CNS-OT index, namely: **Z** **=** **[ln(K**^**0.5**^**) -9.63]/2.02**. For diving in general we suggested a 1% risk, which is close to current U.S. Navy limits (U. S. Department of the Navy, 1991), with the CNS-OT index K not exceeding 26,108 (Arieli, 2019).

## Acclimatization to Oxygen

Oxygen dives were performed by either novice or experienced oxygen divers. We later found that it was possible to divide the novice closed-circuit oxygen divers into two groups: non-sensitive to oxygen (68%) and sensitive to oxygen (32%) (Arieli et al., 2006). The non-sensitive group did not suffer any symptoms of CNS-OT, whereas sensitive divers suffered symptoms of CNS-OT in their initial sequence of dives. In a sample of 50 divers out of 473, of the 16 sensitive divers, 7 suffered CNS-OT only on their first dive. Two suffered CNS-OT on their first 2 dives, but had no symptoms after this. Six suffered CNS-OT on their first 3 dives, but with no further events. One diver suffered CNS-OT from dive 2 through dive 7, though not on his first dive and not subsequent to his seventh. The mean number of dives on which sensitive divers suffered CNS-OT at the start of their diving career was 2.2.

None of the sensitive divers suffered symptoms of CNS-OT on their subsequent dives, despite the fact that these were performed to a greater depth and were longer in duration than their initial, symptomatic dives. This is a clear indication of acclimatization to oxygen after the first few dives. Acclimatization to oxygen has also been suggested by Alcaraz-García et al. (2008), who demonstrated changes in antioxidant levels and a decrease in the amount of damage by reactive oxygen species in closed-circuit oxygen divers following 6 and 12 weeks exposure. Nothing appears to be known about deacclimatization. Thus, there is a process of acclimatization, and possibly also of deacclimatization to oxygen, which makes it necessary to conduct a review of the safe exposure limits.

## Suggestions for Further Research

The effect of acclimatization to hyperoxia on susceptibility to CNS-OT was clearly demonstrated in our previous report (Arieli et al., 2006). We firmly believe that there must also be a deacclimatization mechanism, although we are unaware of any such study. This issue therefore remains open for further investigation in divers who resume oxygen diving after prolonged abstention from hyperoxic exposure. Acclimatization may be related to a decrease in the neuronal calcium ion overload that enhances NO production and neuronal excitotoxicity (Wang et al., 1998). It may additionally be related to reduced activity of eNOS and nNOS, which have been shown to enhance CNS-OT (Demchenko et al., 2003). These mechanisms can be studied further in acclimatized rats or mice.

## Novice and Acclimatized Divers

**Figure 1:**
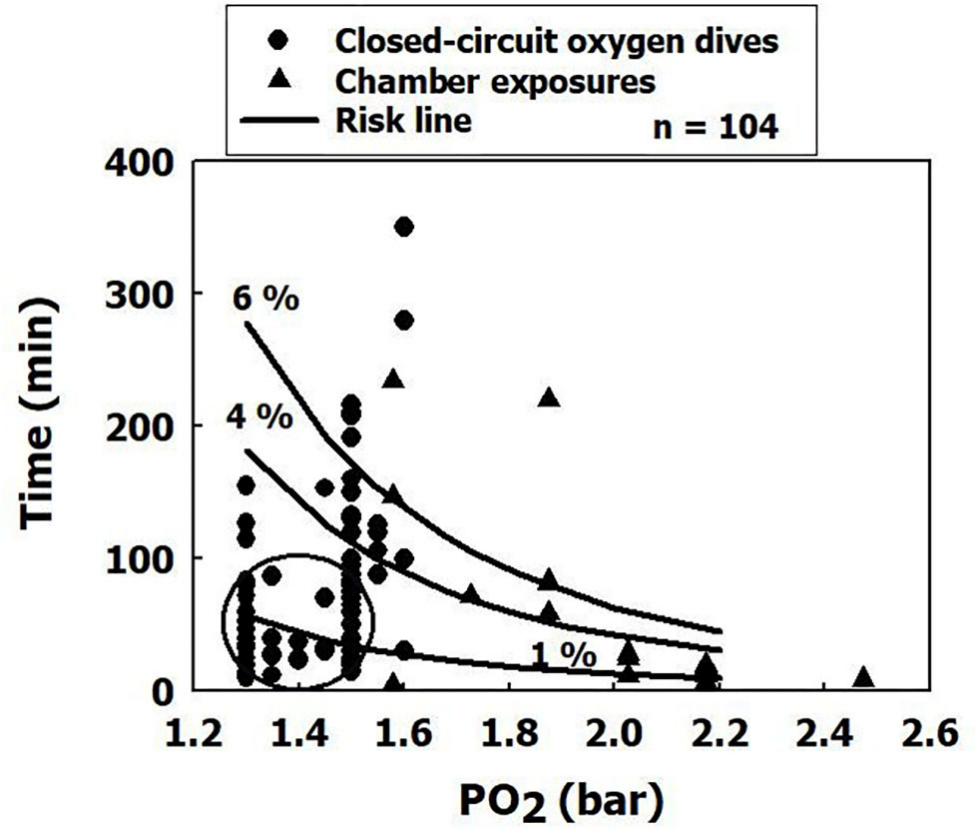
Time/PO_2_ for dives in which there were symptoms of CNS-OT. The ellipse most probably encircles the first few dives (shallower and shorter in duration) of non-acclimatized divers. The risk curves are calculated from the power equation.

We compiled our data for dives with symptoms of CNS-OT from the total data used for the development of the power equation (Arieli et al., 2002), plotting time against PO_2_ in Figure 1. Unfortunately, due to these data having been amassed 24 years previously and recorded on an obsolete system, we were unable to determine which were the data from the first few dives performed by the novice divers. Because the initial closed-circuit oxygen dives were shorter in duration and made to a shallower depth, we encircled those we believed to be their initial dives. In accordance with the risk curves, we suggest that divers who are non-acclimatized to oxygen adhere to the 1% risk level we suggested previously (Arieli, 2019), for which the CNS-OT index should not exceed 26,108. However, in the case of divers acclimatized to oxygen (nitrox, closed-circuit oxygen rebreathers), we would suggest keeping to the limits for a 4% risk, for which the CNS-OT index should not exceed 196,811. This does not entail an actual 4% risk, but rather takes acclimatization into account and introduces a compensatory mechanism. This is the best model science can generate at this moment with the limited data available.

## Author Contributions

All authors listed have made a substantial, direct and intellectual contribution to the work, and approved it for publication.

## Conflict of Interest

The authors declare that the research was conducted in the absence of any commercial or financial relationships that could be construed as a potential conflict of interest.

## Abbreviations

CNS-OT: central nervous system oxygen toxicity
eNOS: endothelial nitric oxide synthase
MET: metabolic equivalent of task
nNOS: neuronal nitric oxide synthase
NO: nitric oxide
PO_2_: partial pressure of oxygen.
